# Systems Drug Discovery for Diffuse Large B Cell Lymphoma Based on Pathogenic Molecular Mechanism via Big Data Mining and Deep Learning Method

**DOI:** 10.3390/ijms23126732

**Published:** 2022-06-16

**Authors:** Shan-Ju Yeh, Tsun-Yung Yeh, Bor-Sen Chen

**Affiliations:** Laboratory of Automatic Control, Signal Processing and Systems Biology, Department of Electrical Engineering, National Tsing Hua University, Hsinchu 30013, Taiwan; m793281@gmail.com (S.-J.Y.); qpctotca@gmail.com (T.-Y.Y.)

**Keywords:** diffuse large B cell lymphoma (DLBCL), deep neural network, drug discovery, drug combination

## Abstract

Diffuse large B cell lymphoma (DLBCL) is an aggressive heterogeneous disease. The most common subtypes of DLBCL include germinal center b-cell (GCB) type and activated b-cell (ABC) type. To learn more about the pathogenesis of two DLBCL subtypes (i.e., DLBCL ABC and DLBCL GCB), we firstly construct a candidate genome-wide genetic and epigenetic network (GWGEN) by big database mining. With the help of two DLBCL subtypes’ genome-wide microarray data, we identify their real GWGENs via system identification and model order selection approaches. Afterword, the core GWGENs of two DLBCL subtypes could be extracted from real GWGENs by principal network projection (PNP) method. By comparing core signaling pathways and investigating pathogenic mechanisms, we are able to identify pathogenic biomarkers as drug targets for DLBCL ABC and DLBCL GCD, respectively. Furthermore, we do drug discovery considering drug-target interaction ability, drug regulation ability, and drug toxicity. Among them, a deep neural network (DNN)-based drug-target interaction (DTI) model is trained in advance to predict potential drug candidates holding higher probability to interact with identified biomarkers. Consequently, two drug combinations are proposed to alleviate DLBCL ABC and DLBCL GCB, respectively.

## 1. Introduction

Non-Hodgkin lymphoma (NHL), a lymphoid tissue malignancy, is one of the most prevalent cancers worldwide [1]. Diffuse large B-cell lymphoma (DLBCL) is the most common subtype of NHL in western countries [2]. Meanwhile, it is a biologically heterogeneous and aggressive disease. The survival rate is usually less than one year for patients without treatment. Along with the thriving of DNA array technology, gene expression profiling studies have confirmed the existence of DLBCL subtypes involving in germinal center B cells (GCB) DLBCL and activated B cells (ABC) DLBCL. It represents lymphomas caused at different stages of lymphatic differentiation. Moreover, DLBCL GCB is a lymphocyte from the germinal center, therefore, it expresses some genes often observed in germinal center B cells including BCL6 and CD10 [3]. The main pathological feature of DLBCL ABC is the NFκB signaling pathway resulting in significant impacts on the cell proliferation and the regulation of apoptosis. It is noted that there is a large difference between DLBCL GCB and DLBCL ABC in terms of the clinical survival rate. The five-year survival rate of DLBCL GCB is about 60%, while the five-year survival rate of DLBCL ABC is about 35%. The pathogenesis of DLBCL in two subtypes is currently unknown.

The current standard therapy for DLBCL is R-CHOP, including five drugs, rituximab, cyclophosphamide, doxorubicin, vincristine, and prednisone. Among them, rituximab acts with CD20 to drive caspase-independent cell apoptosis death [4]. However, rituximab-induced hypogammaglobulinemia occurred [5]. For cyclophosphamide, it could target the gene CD95 and trigger activation-induced cell death after activation [6]. One study indicates that patients treated with cyclophosphamide have a 4.5-fold increased risk of bladder cancer [7]. Doxorubicin, an anthracycline drug, has been implicated in cardiotoxicity. Its main mechanisms have something to do with DNA damage, membrane damage, oxidative stress, and the apoptosis pathways [8]. Targeting the p53 gene to participate in cell cycle arrest, DNA repair, or apoptosis [9], vincristine is widely used to treat malignant tumors; however, vocal cord paralysis caused by neurotoxicity has been found [10]. Prednisone, a glucocorticoid drug, inhibits NFκB and other inflammatory transcription factors, while the long-term steroid therapy may induce osteoporosis and liver cancer [11,12]. Instead of using rituximab, novel anti-CD20 agents (i.e., obinutuzumab and ofatumumab) were suggested for B-chronic lymphocytic leukemia and follicular lymphoma as well [13]. In addition, several innovative treatments for DLBCL have been approved by the U.S. Food and Drug Administration (FDA) including the anti-CD79b antibody drug conjugate polatuzumab vedotin (Pola) with bendamustine and rituximab (Pola-BR) [14]; the oral nuclear transport (XPO1) inhibitor selinexor [15]; and the combination of the anti-CD19 monoclonal antibody tafasitamab with the immunomodulatory agent lenalidomide [16,17]. Considering the different side effects of current treatments, drug combinations with multi-targets therapies toward DLBCL are worth studying.

It usually takes more than 12 years to develop a novel drug. The average cost of the drug development is about USD 2.6 billion [18]. There are few drugs that start from actual human testing that ever make it to marketing [19]. Due to huge demand for new anticancer drugs and various combinations of cell-target based screenings [20], drug repositioning based on computational methods has become popular in drug discovery. Drug-target interaction (DTI) prediction facilitates the process of drug discovery. It is the exploration of new drugs that interact with a particular target. The computational methods for DTI can be broadly classified into ligand-based approaches, docking approaches, and chemogenomic approaches [21]. The concept of ligand-based approaches is to predict the interactions based on the similarities between the protein ligands. However, without using sequencing information, it is hard to discover possible novel interactions due to the limitation of known ligands and protein families [22]. Utilizing 3D structures of proteins as well as drugs, docking approaches are based on the simulations to predict DTI [23,24,25], while these tasks would be challenged for certain membrane proteins, the 3D structures of which are unavailable. For chemogenomic approaches, it combines the chemical space of drugs and the genomics space of proteins into feature vectors to overcome the drawbacks of ligand-based and docking approaches. Chemogenomic approaches is suitable for machine learning (ML) methods for prediction of DTI [26]. In ML methods, the knowledge about drugs and proteins are represented by feature vectors that are used to train models for predicting the interactions between new drugs and/or new targets [27]. Furthermore, different learning-based models have been developed for DTI predictions, such as deep belief neural networks [28,29], convolutional neural networks [30,31], multilayer perceptrons [32,33], and graph neural network [34,35,36]. From the viewpoint of application, taking advantage of a chemogenomic approach, we trained a deep neural network (DNN)-based DTI prediction framework in advance for obtaining potential drug candidates toward the identified biomarkers.

**Figure 1:**
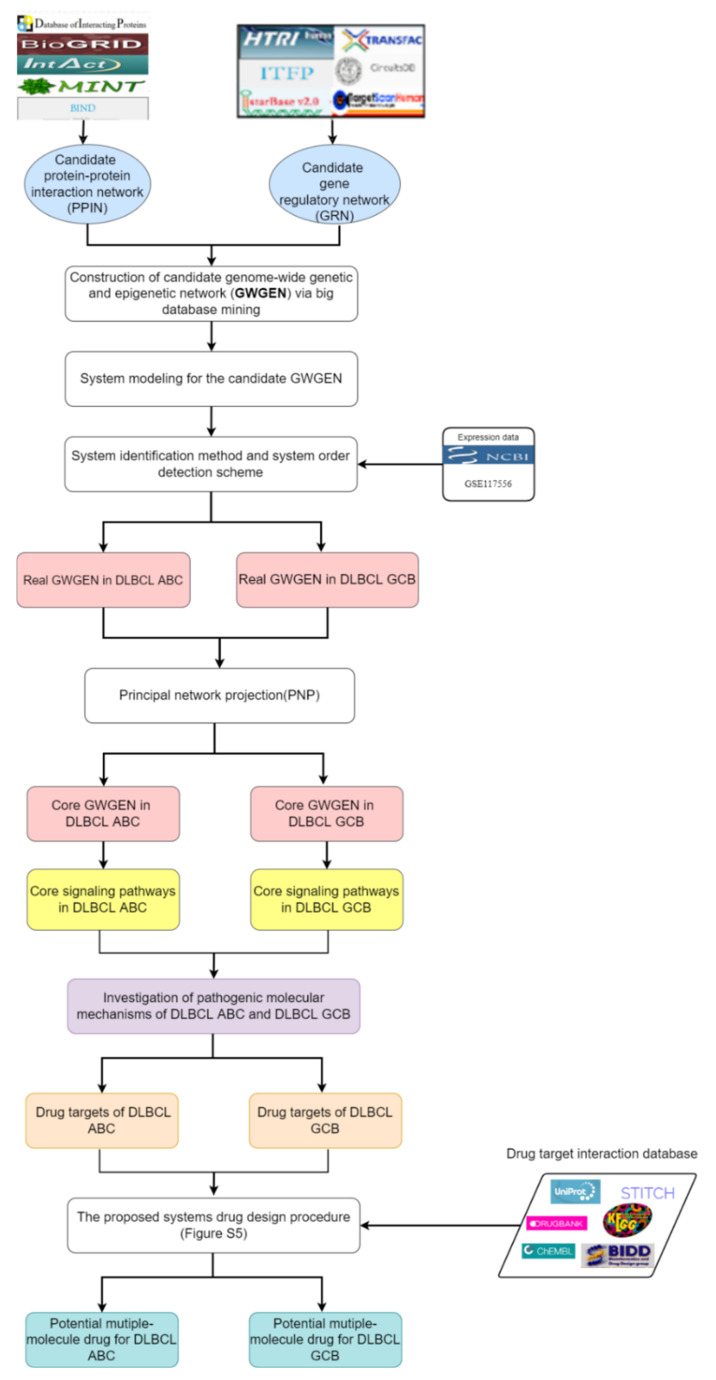
Flowchart of systems drug discovery based on systems biology approaches and drug design specifications.

In this study, we propose systems biology methods including systems modeling, system identification, system order detection scheme, and a principal network projection method to identify essential biomarkers as drug targets based on investigating pathogenic molecular mechanisms. Afterward, for identified biomarkers, we follow system drug design procedure taking drug design specifications into account, such as drug-target interaction ability, drug regulation ability, and drug toxicity to suggest potential drug combinations for DLBCL GCB and DLBCL ABC, respectively. The corresponding systems drug discovery flowchart is shown in Figure 1. It is noted that we build a DNN-based DTI model in advance for helping us obtain drug candidates, which have higher interaction probability toward the identified biomarkers (drug targets). Consequently, both famotidine and chlorzoxazone are regarded as common molecular drugs, which contribute to inhibiting tumor metastasis, migration, and invasion for DLBCL ABC and DLBCL GCB. Furthermore, etoposide is designed specifically for cancer cell DNA damage of DLBCL ABC, and methotrexate is designed specifically for abnormal cell cycle of DLBCL GCB.

## 2. Results

### 2.1. The Pathogenic Molecular Mechanisms in DLBCL ABC

**Figure 2:**
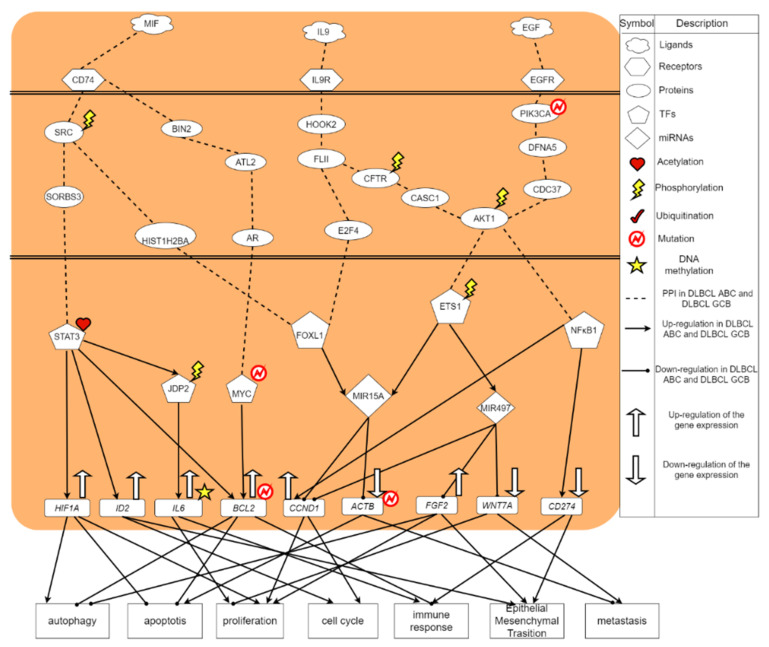
The core signaling pathways of DLBCL ABC. The black dotted line indicates protein–protein interactions in DLBCL ABC; the black arrow head of solid lines means activating cellular functions; the black circle head of solid lines means inhibiting cellular functions; the up arrow on the target gene indicates a high expression. The down arrow on the target gene indicates a low expression.

From the core signaling pathways of DLBCL ABC in Figure 2, macrophage migration inhibitory factor (MIF) is found to be an important regulator of the innate immune system. MIF is classified as a pro-inflammatory cytokine [37]. MIF binds to CD74 on other immune cells to trigger an acute immune response [38]. Receptor CD74 (HLA class II histocompatibility antigen γ-chain receptor) in DLBCL ABC receives microenvironment factor MIF (macrophage migration inhibitory factor) to regulate TF STAT3 and MYC [39], respectively. The signaling transduction protein SRC, which was affected by phosphorylation, could transmit signals from CD74 to TF STAT3 in DLBCL ABC. Moreover, SRC was phosphorylated at the specific tyrosine residue by other tyrosine kinases, playing an important role in regulating embryonic development and cell growth [40]. Another signaling transduction protein, SORBS3, encodes a SH3 domain-containing adaptor protein. The presence of the SH3 domain is responsible for making the protein bind other cytoplasmic molecules, which are helpful for cytoskeletal organization, cell migration, gene expression and signaling. The constitutive activation of STAT3 signal promotes the growth, survival, angiogenesis and metastasis of tumor cells [41]; the overexpression of abnormally acetylated (activated) TF STAT3 can upregulate its target gene *HIF1A* [42], thereby promoting cellular functions, including cell proliferation as well as autophagy and inhibiting apoptosis [43]. At the same time, TF STAT3 would upregulate the target gene *ID2* [44] resulting in the promotion of cell cycle and epithelial-mesenchymal transition (EMT). Besides, it would upregulate the target gene *BCL2*, triggering the inhibition of autophagy and apoptosis. Upregulated by the acetylated STAT3, TF JDP2 is related to the inhibition of cell differentiation, cell cycle, and apoptosis. After being modified by the phosphorylation, the activated TF JDP2 would upregulate the DNA-methylated target gene *IL6*, which leads to promoting cell apoptosis and immune response against cancer [45].

Increased expression of SRC would trigger another core signaling pathway transmitting signals to TF FOXL1 through signaling transduction protein HIST1H2BA. FOXL1 plays an important role in regulating the expression of genes involved in cell metabolism, proliferation and differentiation. The overexpression of FOXL1 can upregulate miRNA MIR15A. The overexpression of MIR15A awakened by the upstream signals would inhibit the target genes *CCND1* and *ACTB* to promote their respective cellular functions. However, the total expression of *CCND1*, which is also activated by another TF NFκB1 and miRNA MIR497, is upregulated. Moreover, the target gene *CCND1* can promote the cell cycle progression and the target gene *ACTB* can promote the cell apoptosis and metastasis.

In the next core signaling pathway, after the ligand MIF combining with the receptor CD64, the signals are transmitted to TF MYC via the signaling transduction proteins BIN2, ATL2, and AR in DLBCL ABC. It is known that BIN2 related pathways are immune system [46]. Moreover, it can facilitate cell movement and migration through podosomes that interact with cell membrane and mediate cytoskeleton. Among this core signaling pathway, AR, an androgen receptor, is a DNA-binding transcription factor that regulates gene expression of *BCL2* [47]. It can regulate gene expression in eukaryotes and affect cell proliferation and differentiation. MYC is a proto-oncogene, which plays an important role in cell cycle progression, apoptosis and metastasis [48]. Overexpressed TF MYC will promote the upregulation of the target gene *BCL2*, further inhibiting cell autophagy and apoptosis, and promoting immune response.

### 2.2. The Carcinogenic Molecular Mechanism in DLBCL GCB

**Figure 3:**
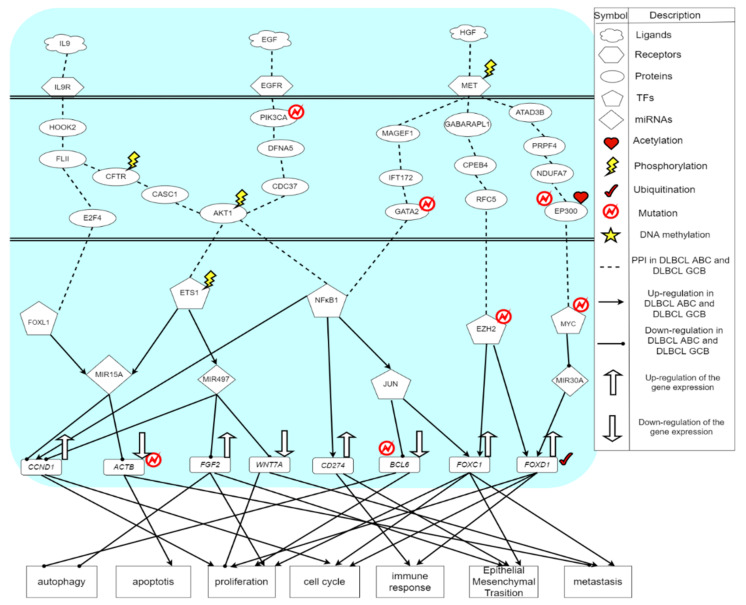
The core signaling pathways of DLBCL GCB: The dotted black line indicates protein–protein interactions of DLBCL GCB; the black arrow head of solid lines means activating cellular functions; the black circle head of solid lines means inhibiting cellular functions; the up arrow on the target gene indicates an up-regulation. The down arrow on the target gene indicates a down-regulation.

The core signaling pathways of DLBCL GCB are shown in Figure 3. The microenvironment factor is a hepatocyte growth factor (HGF). HGF is secreted by mesenchymal cells and acts as a multifunctional cytokine on cells of primary epithelial origin [49]. Its ability to stimulate mitosis, cell movement and cytoplasmic matrix invasion makes it angiogenic, and plays a significant role in tumorigenesis and tissue regeneration [50]. The tyrosine kinase receptor MET receives the microenvironment factor HGF to regulate TF NFκB1, EZH2 and MYC, respectively. MET is an essential tyrosine kinase receptor for embryonic development, organ growth and wound healing. Through the signaling transduction proteins MAGEF1, IFT172 and GATA2, the mutated GATA2 protein will transmit the signal from MET to TF NFκB1. Among them, MAGEF1 can promote the degradation of proteasome and weaken the activity of some DNA repair and metabolic enzymes. In order to form cilia, IFT is necessary for the movement of other signaling proteins in the cilia [51]. Therefore, IFT172 plays a role in many different signaling pathways. IFT is considered to be a mediator of Hedgehog signaling and is one of the most important pathways in embryogenesis. Furthermore, GATA2 plays an important role in regulating the transcription of genes related to the development and proliferation of hematopoietic and endocrine cells [52]. The mutation of GATA2 is associated with a variety of genetic and immune diseases, including myelodysplastic syndrome and acute myeloid leukemia [53]. The overexpressed NFκB1 can upregulate TF JUN. An improper activation of NFκB is related to many inflammatory diseases, while continuing to inhibit NFκB can cause abnormal immune cell development or delayed cell growth. This signal transduction event can lead to many biological processes such as inflammation, immunity, differentiation, cell growth, triggering growth, tumorigenesis and apoptosis. TF JUN was found to play an important role in cell proliferation [53]. In DLBCL GCB, TF JUN will downregulate the target gene *BCL6* and upregulate the target gene *FOXC1*, thereby correspondingly resulting in cell proliferation, autophagy, cell cycle, epithelial-mesenchymal transition (EMT) and cell metastasis.

In addition, after receiving the signal from phosphorylated MET, the signaling transduction proteins GABARAPL1, CPEB4, and RFC5 transmit the signal to TF EZH2. GABARAPL1 is a protein related to autophagy [54]. CPEB4 is related to the cell cycle progression promoting the growth and proliferation of tumors [55]. RFC5 is involved in DNA replication and repair. TF EZH2 is responsible for healthy embryo development through the epigenetic maintenance of genes, which take charge of regulating development and differentiation [56]. The mutation or overexpression of EZH2 is associated with a variety of cancers [57]. Blocking the activity of EZH2 may slow down tumor growth. It is known that EZH2 has become a target for inhibition as it was upregulated in a variety of cancers [58]. In Figure 2, the abnormally activated TF EZH2 can upregulate target gene *FOXC1*, further promoting cellular functions, including cell proliferation, cell cycle, epithelial-mesenchymal transition (EMT) and metastasis [59]. In addition, EZH2 will also upregulate *FOXD1*, causing cell proliferation, cell cycle and immune response.

In the next core signaling pathway, after HGF binding to MET, the signal will be transmitted to TF MYC via the signaling transduction proteins ATAD3B, PRPF4, NDUFA7, and EP300, where ATAD3B is a protein related to immunity, PRPF4 is involved in pre-mrna splicing and modification [60], and NDUFA7-related pathways include respiration electronic transportation [61]. The mutant protein EP300 affected by acetylation can promote the transmission of upstream signals to downstream regulators. EP300 plays an important role in regulating cell growth and division, and promotes cell maturation and differentiation. One study indicates that EP300 protein is crucial for normal development of multicellular organisms before and after birth [62]. The expression of EP300 in DLBCL is significantly reduced. The mutations in EP300 usually remove or inactivate the histone acetyltransferase (HAT) coding domain of any gene [63]. In addition, study has pointed out that TF MYC was usually expressed constitutively in cancer [64], which led to an increased expression of many genes, some of which were involved in cell proliferation, in turn leading to cancer formation. The abnormally activated TF MYC will silence miRNA MIR30A [65]. Moreover, the low expression of MIR30A can negatively regulate the target gene *FOXD1*, thereby promoting cell proliferation, cell cycle and immune response.

Finally, from the results shown in Figure 1 and Figure 2, although the cancer cells of DLBCL GCB have a stronger proliferative ability than the cancer cells of DLBCL ABC, we find that the ability of anti-apoptosis in DLBCL GCB is worse than DLBCL ABC. As a result, the cancer cells of DLBCL GCB are not conducive to spreading to other cell tissues. In other words, with stronger anti-apoptosis and anti-immune ability, DLBCL ABC will possess excessive cancer cell proliferation, which enhance the effect of metastasis and EMT. Therefore, DLBCL ABC has a higher mortality rate than GCB subtype.

### 2.3. The Common and Specific Carcinogenic Molecular Mechanism between DLBCL ABC and DLBCL GCB

**Figure 4:**
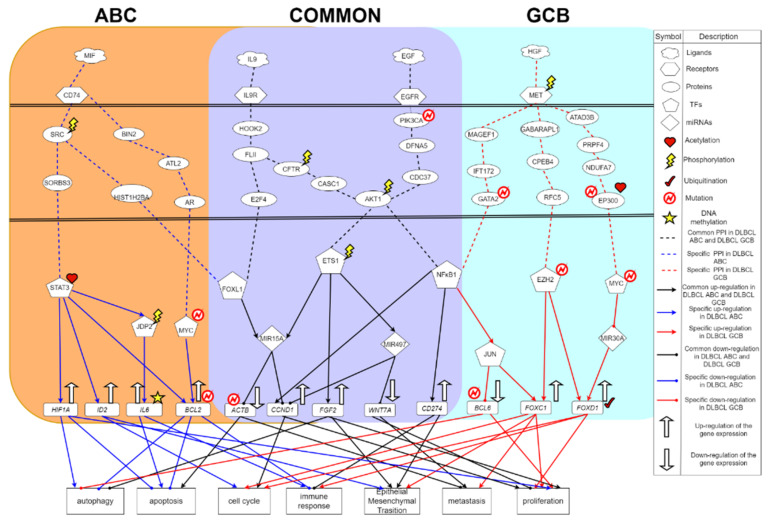
The common and specific core signaling pathways of DLBCL ABC and DLBCL GCB. This figure summarizes the genetic and epigenetic pathogenic molecular mechanisms of DLBCL ABC and DLBCL GCB. The core signaling pathways shown in the purple background are the common core signaling pathways of DLBCL ABC and DLBCL GCB. The blue line indicates specific core signaling pathways of DLBCL ABC; the red line indicates specific core signaling pathways of DLBCL GCB; the black line indicates common core signaling pathways of DLBCL ABC and DLBCL GCB; the arrow head of solid lines means activating cellular functions; the circle head of solid lines means inhibiting cellular functions. The up arrow on the target gene indicates an up-regulation. The down arrow on the target gene indicates a down-regulation.

In Figure 4, we have investigated the common and specific core signaling pathways between DLBCL ABC and DLBCL GCB. The microenvironment factor IL9 is a cell growth factor that can stimulate cell proliferation and prevent apoptosis [66]. Interleukin 9 receptor (IL9R) accepts IL9, a pleiotropic cytokine, belonging to the group of interleukins, through the signaling transduction proteins HOOK2, FLII, E2F4 in DLBCL ABC and DLBCL GCB, to upregulate TF FOXL1. In this core signaling pathway, the transduction signaling protein FLII plays a role in regulating cytoskeletal rearrangement involved in cell division and cell metastasis; E2F4 plays an important role in controlling the cell cycle and inhibiting tumor proteins. TF FOXL1 would regulate the expression of genes involved in cell metabolism, proliferation and differentiation [67].

The upregulated TF FOXL1 will overexpress MIR15A while MIR15A negatively regulates target genes *CCND1* and *ACTB*, respectively [68]. There have been studies showing that ACTB will mutate in DLBCL [69]. As for *CCND1*, it will cause cell proliferation and cell cycle progression [70]. After DNA replication, the replication chromosomes are separated into two independent cells. Generally speaking, the cell cycle can be divided into interphase (I) and mitosis (M). The stages of mitosis include prophase, prometaphase, metaphase, anaphase and telophase. The interphase (phase I) can usually be divided into the early stage of DNA synthesis (G1), the period of DNA synthesis (S) and the late stage of DNA synthesis (G2) [71]. The entire cell cycle can be expressed as: G1 phase → S phase → G2 phase → M phase. In G1 phase, the G1 checkpoint mechanism will prepare to ensure DNA synthesis. Once the cell cycle checkpoint (Start or Restriction Point) is passed, the cell cycle is initiated and the process is irreversible, in which CCND1 is the most important cell cycle checkpoint [72]. DNA replication occurs in the S phase. During G2, the cells will prepare for mitosis, but in some cases the cells will jump out of the cell cycle and enter the so-called G0 phase. In G0 phase, the cells will leave the cycle and stop dividing. In fact, many cells in the human body are usually in the G0 phase, for example, nerve cells will never divide. The downregulation of ACTB will inhibit cell apoptosis and promote cell metastasis. Therefore, this core signaling pathway leads to the proliferation, the anti-apoptosis and the promotion of metastasis to exacerbate cancer progression in DLBCL patients. In addition, the receptor IL9R also transmits signals to TF ETS1 through signaling transduction proteins HOOK2, FLII, CFTR, CASC1, AKT1, where CFTR (cystic fibrosis transmembrane conductance regulator) is a membrane protein and chloride channel in vertebrates, which transport negatively charged particles called chloride ions into or out of the cell [73].

For the next core signaling pathway in Figure 4, the epidermal growth factor receptor (EGFR) receives the microenvironmental factor EGF, and then the signal is transmitted through the transduction proteins via the mutated PIK3CA, DFNA5, CDC37, and the phosphorylated AKT1 to TF ETS1, in which AKT1 is related to apoptosis [74]. It is found that AKT1 will phosphorylate AKT and inhibit apoptosis. ETS1 is a TF related to maintaining the proliferation of DLBCL and regulating the differentiation of germinal centers [75]. The overexpression of ETS1 will cause proliferation, survival and differentiation of lymphoma cells [76]. Modified by phosphorylation, the TF ETS1 will promote the expression of MIR15A and MIR497; meanwhile, ETS1 will upregulate the target gene FGF2. Furthermore, MIR497 will inhibit *CCND1* and *WNT7A*. It is known that *WNT7A* will promote cell proliferation and metastasis [77]. MIR15A will inhibit *CCND1* and *ACTB*. Although *CCND1* is inhibited by MIR15A and MIR497, *CCND1* is upregulated by another TF like NFκB1 as well. Hence, the total expression of *CCND1* in DLBCL is still upregulated, leading to subsequent cell proliferation and metastasis. In addition, *FGF2* can cause the inhibition of autophagy [78]. Autophagy is an orderly cell degradation and recycling process in all eukaryotes. There are generally three different forms of autophagy, including microautophagy, macroautophagy, and chaperone-mediated autophagy (CMA) [79]. One of their functions is to transport the cargo to the lysosome for degradation and recycling. *FGF2* will also promote cell proliferation and epithelial-mesenchymal transition (EMT). Therefore, this corresponding core signaling pathway will cause cell proliferation; simultaneously, autophagy and apoptosis will be inhibited, which further promote cancer cell metastasis and EMT in DLBCL. Moreover, the formation process of EMT will destroy the adhesion between normal cells improving the ability of migration and invasion for the cancer cells. It brings benefit to the cancer metastasis. This process not only accelerates the spread of cancer cells but also makes cancer cells spread intensely [79].

The microenvironment factor EGF (epidermal growth factor) plays an important role in regulating cell growth, proliferation and differentiation. After EGF binding to the receptor EGFR on the cell surface, the signals would be transmitted to TF NFκB1 through the signaling transduction proteins including the mutated PIK3CA, DFNA5, CDC37, and the phosphorylated AKT1. The role of PIK3CA is to promote the catalytic reaction of the message transmission [80]; and the mutation of PIK3CA will change the way of cells, regulating physiological responses to cause the formation of cancer. DFNA5 also has been found in other types of cancer such as stomach cancer, colorectal cancer, and breast cancer. Its characteristic is to induce apoptosis. Among this pathway, CDC37, a molecular chaperone protein, has a specific function in cell signaling transduction. It binds to a variety of kinases and regulates cyclin. Moreover, the TF NFκB1 will promote the upregulation of the target gene *CCND1*. Improper activation of NFκB is related to many inflammatory diseases, and continuous inhibition of NFκB will lead to poor development of immune cells or the delay of cell growth [81]. In summary, this signal transduction event is associated with many biological processes, such as inflammation, immunity, differentiation, and cell growth. It finally triggers cell growth, tumorigenesis and apoptosis. Besides, the upregulated *CCND1* further promotes cellular functions including cell proliferation and cell cycle progression. TF NFκB1 also upregulates the expression of target gene *CD274* (*PD-L1*), which in turn triggers cellular functions of EMT and immune responses [82].

### 2.4. Systems Drug Design Procedure Considering Drug-Target Interaction, Drug Regulation Ability, and Drug Toxicity

After investigating pathogenic molecular mechanisms, we identified two pools of essential biomarkers as drug targets for two subtypes of DLBCL shown in Table 1. The systems drug design procedure is in Figure S5. Firstly, we consider the drug-target interaction ability toward the identified biomarkers in terms of the application of DNN-based DTI model. Subsequently, filtered by drug regulation ability and drug toxicity, the number of predicted drug candidates would be narrowed down. For training DNN-based DTI model, there were 70% of the data as training set, including 10% of the data as validation set. The remaining 30% of the data were used as the testing set. To the architecture of DNN-based DTI model, it is a fully connected neural network consisting of one input layer, four hidden layers and one output layer, of which four hidden layers have 512, 256, 128, 64 neurons, respectively. The dropout was added in each hidden layer for reducing overfitting. We used ReLU as the activation function for each hidden layer. In the output layer, we chose sigmoid to be the activation function for limiting the output value between zero and one. It is noted that the drugs with higher interaction probability (greater than 0.5) would be selected as drug candidates. Evaluating the robustness of hyperparameters including the number of nodes, dropout, and learning rate, we performed 10-fold cross validation. The corresponding 10-fold cross validation results could be found in Figure S6. The average accuracy of testing is 98.698% (standard deviation: 0.0659). Furthermore, we plot the receiver operating characteristic curve (ROC) in Figure S7. The area under ROC of the DNN-based DTI model is 0.99. Here, except for drug-target interaction, we regard drug regulation ability and drug toxicity as our drug design specifications as well. By referring to the connectivity map (CMap) [83], we could find the gene signatures after treating with more than 1300 compounds in numbers of cultivated cell lines. The goal here is to find the drugs owning the ability to reverse the abnormal gene expression. Meanwhile, according to the median lethal dose, which is looked up at DrugBank [84], we expect that the selected candidate small molecules could have less toxicity (Table S4). Consequently, we suggested famotidine, chlorzoxazone, and etoposide to be the potential multiple-molecule drug for alleviating DLBCL ABC (Table 2); famotidine, chlorzoxazone, and methotrexate as potential multiple-molecule drug for mitigating DLBCL GCB (Table 3).

## 3. Discussion

Based on the core signaling pathways, we investigated the downstream carcinogenic pathogenesis and identified five significant biomarkers as drug targets for DLBCL ABC and DLBCL GCB, respectively (Table 1). Among these biomarkers, STAT3 and MYC can influence cancer cell survival and promote proliferation. The immune response of a human can be inhibited by NFκB1. Both AKT1 and EZH2 are associated with cancer metastasis and invasion, resulting in the deterioration of tumors. In contrast, FOXL1 can promote apoptosis and inhibit cancer cell metastasis. In order to reduce the ability of inhibiting apoptosis and promoting proliferation by HIF1A, to diminish the ability of promoting proliferation and cell cycle by ID2, to decrease the ability of reducing apoptosis, and to reduce the ability of inhibiting autophagy and suppressing immunity by BCL2, STAT3 was selected as the biomarker to be inhibited. For enhancing the ability of promoting apoptosis and inhibiting metastasis caused by the target gene ACTB, FOXL1 was selected as a biomarker to be up-regulated. Moreover, to reduce the ability of inhibiting autophagy, promoting proliferation and EMT by FGF2, and to inhibit the ability of promoting cancer cell metastasis by WNT7A, AKT1 was selected as a biomarker to be inhibited. In addition, considering the significant impact of immune response on DLBCL ABC and GCB, NFκB1 was selected as the drug target to be inhibited, thereby reducing the ability of both suppressing immune response by CD274 and promoting cell cycle and proliferation by CCND1. For the purpose of reducing the ability of promoting proliferation, metastasis, and EMT by FOXC1 and reducing the proliferation caused by FOXD1, EZH2 was selected as a biomarker to be down-regulated.

Using immunotherapy against cancer gains a lot of attention in recent years. Here, we selected NFκB1 as a drug target to indirectly inhibit PD-L1 and block the related mechanisms having contribution to escape immunity. Among the proposed two multiple-molecule drugs, chlorzoxazone is a drug for treating muscle spasms [85]. It acts on the spinal cord by suppressing reflexes. One tumor related study has shown that it was used with other drugs to inhibit tumor growth, including tumor metastasis, migration, and invasion [86]. It is known that STAT3 and NFκB1 are significant activators of carcinogenic signal transduction. Chlorzoxazone can effectively reduce the expression of STAT3, NFκB1, and EZH2, and upregulate FOXL1, therefore, it might be an effective drug for DLBCL. In addition, famotidine could decrease the production of stomach acid. Its pharmacologic activity is used in the treatment of acid-related gastrointestinal conditions, including duodenal ulcer, esophageal adenocarcinoma and chronic gastroesophageal reflux disease in adults and children. Meanwhile, famotidine can inhibit the occurrence of cancer by inhibiting STAT3. Its drug targets include NFκB1, AKT1, and EZH2 as well. Moreover, etoposide has been studied to replace the DLBCL current standard treatment with R-CHOP, including rituximab, cyclophosphamide, doxorubicin, vincristine and prednisone, among them, the disadvantage of doxorubicin is its cardiotoxicity [87]. Another study also mentioned that etoposide substituted could treat most of DLBCL patients who cannot receive anthracycline treatment [88]. Methotrexate, a chemotherapeutic drug and immunosuppressive agent, has been commonly applied in combination with other drugs for the treatment of breast cancer, leukemia, lung cancer, lymphoma, autoimmune disease, and ectopic pregnancy [89]. By down-regulating its target protein EZH2, methotrexate can treat cancers [89]. Note that dysregulation of EZH2 is closely related to oncogenesis of various tissue types. More and more evidences show that targeting EZH2 has great therapeutic potential in cancers [90]. Methotrexate can not only downregulate the expression of EZH2, but also interact with AKT1 and MYC. Hence, we suggest it as one of the small molecule drugs in our proposed drug combination.

Given the substantial costs and long development timeline of new drug discovery, the repurposing of old drugs to treat common and rare disease becomes an attractive proposition. In other words, drug repurposing is a strategy for identifying new uses for approved drugs that are outside the scope of known medical indication. In this study, by the proposed systems biology approaches and drug design specifications, we suggested multiple-molecule drugs (drug combinations) for DLBCL ABC and GCB, respectively. Those suggested small molecules are FDA approved. Although some studies have shown that etoposide and methotrexate were used in DLBCL, their combinations with famotidine and chlorzoxazone are still worth studying in the future in terms of synergistic and antagonistic effects. Leveraging computational biology methods, this study might provide new perspectives of understanding the pathogenic molecular mechanisms of DLBCL ABC and DLBCL GCB at a system level and give an alternative way to accelerate systems drug discovery for new therapeutics.

## 4. Materials and Methods

### 4.1. Overview of Systems Drug Discovery for DLBCL ABC and DLBCL GCB

The DLBCL microarray data is from the National Center for Biotechnology Information (NCBI) with accession number GSE117556. The corresponding platform is GPL14951. The dataset samples were divided into two subtypes, DLBCL ABC and DLBCL GCB, in which the ABC subtype has worse prognosis. There are 468 samples and 249 samples for DLBCL GCB and DLBCL ABC, respectively. The flowchart of systems drug discovery is in Figure 1. For identifying essential biomarkers as drug targets to alleviate DLBCL ABC and DLBCL GCB, we investigated the pathogenic molecular mechanisms based on the systems biology methods: (1) big database mining; (2) system modeling; (3) system identification and system order detection scheme; (4) principal network projection method.

Firstly, by big database mining, we constructed a candidate genome-wide genetic and epigenetic network (GWGEN), which is represented by a Boolean matrix (i.e., 0 or 1 if interaction is nonexistent or existent between two nodes). It is noted that both DLBCL ABC and DLBCL GCB shared the same candidate GWGEN. The candidate GWGEN consists of the candidate protein–protein interaction network (PPIN) and candidate gene regulatory network (GRN). For the candidate PPIN, we refer to the following database: DIP [91], IntAct [92], BioGRID [93], BIND [94], and MINT [95]. To the candidate GRN, we collect the pairs of transcription factors and target genes from ITFP [96] and HTRIdb [97]. Moreover, we look up databases including TargetScan [98], CircuitsDB [99], and StarBase2.0 [100] for the post-transcriptional regulations between miRNA, lncRNA and their target genes. After conducting system modeling for proteins, genes, miRNAs, and lncRNAs, we could evaluate system models’ parameters by the system identification method with the help of microarray dataset in two subtypes of DLBCL. There might be false-positive interactions in the candidate GWGEN caused by various experimental conditions. Therefore, we performed a system order detection approach to prune these false-positive interactions for obtaining real GWGENs for DLBCL ABC and DLBCL GCB, respectively (Figures S1 and S2). The total number of nodes (i.e., transcription factors, receptors, proteins, miRNAs, lncRNAs) and their corresponding edges in the candidate GWGEN, real GWGEN of DLBCL ABC, and real GWGEN of DLBCL GCB are shown in Table S1. However, the real GWGENs were still too complicated to analyze. Applying principal network projection (PNP) method, from the real GWGENs, we could extract core GWGENs (Figures S3 and S4), which is comprised of the top 3000 nodes based on the descending order of projection value. The higher the projection value is, the more contribution provided by the node in the real GWGEN. Additionally, we did gene enrichment analyses by the Database for Annotation, Visualization and Integrated Discovery (DAVID) Bioinformatics Resources version 6.8 based on the genes in core GWGENs (Tables S2 and S3). Projecting the corresponding core GWGENs in the annotation of Kyoto Encyclopedia of Genes and Genomes (KEGG), we could further investigate the common and specific pathogenic molecular mechanisms and identify essential biomarkers as drug targets.

For suggesting potential drug candidates toward these identified drug targets, we followed systems drug design procedure shown in Figure S5. The drug design specifications include drug-target interaction probability, drug regulation ability, and drug toxicity. To estimate drug-target interaction probability, we trained a DNN-based DTI model in advance. We regard the drugs having higher predicted probability as drug candidates. Subsequently, the number of those predicted drug candidates would be narrowed down by considering drug regulation ability and toxicity. Here, we aim to find drugs having the ability to reverse abnormal gene expression with low toxicity. More details will be discussed in the following sections.

### 4.2. Constructing the System Models in the GWGEN to Identify Real GWGEN of DLBCL GCB and DLBCL ABC

To investigate the molecular mechanisms of DLBCL GCB and DLBCL ABC, we constructed the interactive and regulatory models in the candidate GWGEN, including protein–protein interactions, transcriptional regulations, miRNA regulations, and lncRNA regulations. For the candidate PPIN (PPIN), the *i*-th protein is described in the following equation:

(1) $$p_{i}\left[n\right]=\sum_{\begin{array}{c} k=1\\ k\neq i \end{array}}^{Y_{i}}\alpha_{ik}p_{i}\left[n\right]p_{k}\left[n\right]+\phi_{i,PPIN}+\eta_{i,PPIN}\left[n\right]\;\mathrm{for}\;i=1,\ldots ,I,\;\mathrm{and}\;n=1,\ldots N.$$

where $\alpha_{ik}$ is the interaction ability between the *i*-th protein and the *k*-th interactive protein; $p_{i}\left[n\right]$ denotes the expression level of the *i*-th protein for the sample *n*; $p_{k}\left[n\right]$ represents the expression level of the *k*-th protein for the *n* data sample; $Y_{i}$ indicates the total number of proteins interacting with the *i*-th protein and *I* denotes the total number of proteins in the candidate PPIN; *N* is the total number of data samples; $\phi_{i,PPIN}$ is the basal level of the *i*-th protein caused by some unknown interactions including phosphorylation and acetylation; $\eta_{i,PPIN}\left[n\right]$ represents the stochastic noise as a result of the modeling residue and measurement noise for the *n* data sample.

For the candidate gene regulatory network (GRN) in the candidate GWGEN, the systematic gene regulation model for the *q*-th gene of DLBCL cells to sample *n* can be governed by the following form:

(2) $$g_{q}\left[n\right]=\sum_{\begin{array}{c} j=1\\ j\neq q \end{array}}^{J_{q}}A_{qj}z_{j}\left[n\right]+\sum_{w=1}^{W_{q}}B_{qw}x_{w}\left[n\right]-\sum_{h=1}^{H_{q}}C_{qh}d_{h}\left[n\right]g_{q}\left[n\right]+\phi_{q}+\eta_{q}\left[n\right]\;\mathrm{for}\;q=1,\ldots ,Q\;\mathrm{and}\;n=1,\ldots N.$$

where $g_{q}\left[n\right]$ represents the expression level of the *q*-th gene; $J_{q}$ indicates the total number of TFs binding to the *q*-th gene; $W_{q}$ represents the total number of lncRNAs binding to the *q*-th gene; $H_{q}$ denotes the total number of miRNAs inhibiting the *q*-th gene; $A_{qj}$ denotes the transcription regulatory ability from the *j*-th TF to the *q*-th gene; $B_{qw}$ is the regulation ability from the *w*-th lncRNA to the *q*-th gene; $C_{qh}\geq 0$ represents the post-transcription regulatory ability, with which the *h*-th miRNA inhibits the *q*-th gene; $z_{j}\left[n\right]$, $x_{w}\left[n\right]$, and $d_{h}\left[n\right]$ indicate the expression of the *j*-th TF, the *w*-th lncRNA, and the *h*-th miRNA, respectively. *Q* is the total number of genes and *N* denotes the total number of data samples; $\phi_{q}$ represents the basal level of the *q*-th gene expression due to unknown regulations containing post-transcriptional modifications; $\eta_{q}\left[n\right]$ is the stochastic noise of the *q*-th gene for the data sample *n* caused by the model uncertainty and data noise. Furthermore, in the same way, the systematic models of the candidate lncRNA regulation network and the candidate miRNA regulation network can be referred to in the Supplementary Materials.

### 4.3. Using the System Identification Method and System Order Detection Approach to Build Real GWGENs of DLBCL GCB and DLBCL ABC

To estimate the unknown parameters for the PPI model in the candidate PPIN, we utilize a system identification method and system order detection approach on our systematic models with the help of genome-wide microarray data of patient samples. The PPI equation in Equation (1) could be rewritten as below:

(3) $$\begin{array}{ll} p_{i}[n] & =\left[p_{i}[n]p_{1}[n]\;\cdots \;p_{i}[n]p_{Y_{i}}[n]\;1\right]\times \left[\begin{array}{c} \alpha_{i1}\\ \vdots \\ \alpha_{iY_{i}}\\ \phi_{i} \end{array}\right]+\eta_{i}\left[n\right]\\ & =\xi_{i}[n]\cdot \varphi_{i,P}+\tau_{i}[n],\;\mathrm{for}\;i=1,\ldots ,I\;\mathrm{and}\;n=1,\ldots ,N. \end{array}$$

where $\xi_{i}[n]$ determines the regression vector, which could be computed by the microar- ray data; $\varphi_{i,P}$ indicates the unknown parameter vector for the *i*-th protein. The Equation (3) of the *i*-th protein could be augmented for *N* samples as below:

(4) $$\left[\begin{array}{c} p_{i}[1]\\ p_{i}[2]\\ \vdots \\ p_{i}[N] \end{array}\right]=\left[\begin{array}{c} \xi_{i,P}[1]\\ \xi_{i,P}[2]\\ \vdots \\ \xi_{i,P}[N] \end{array}\right]\cdot \varphi_{i,P}+\left[\begin{array}{c} \tau_{i}\left[1\right]\\ \tau_{i}\left[2\right]\\ \vdots \\ \tau_{i}\left[N\right] \end{array}\right]$$

Furthermore, the Equation (4) could be simplified represented as:

(5) $$P_{i}=\Xi_{i,P}\cdot \varphi_{i,P}+T_{i}$$

Therefore, the unknown parameters in the vector $\varphi_{i,P}$ could be estimated by solving the least square estimation problem:

(6) $${\hat{\varphi }}_{i,P}=\underset{\varphi_{i,P}}{\min}\frac{1}{2}{\left\|\Xi_{i,P}\cdot \varphi_{i,P}-P_{i}\right\|}_{2}^{2}$$

where ${\hat{\varphi }}_{q,G}$ is the estimated vector including the estimated interaction parameters for the *i*-th protein.

In the same way, the gene regulation model in Equation (2) could be rewritten as below:

(7) $$\begin{array}{ll} g_{q}[n] & =\left[z_{1}[n]\;\cdots \;z_{J_{q}}[n]\;x_{1}[n]\;\cdots \;x_{W_{q}}[n]\;g_{q}[n]d_{1}[n]\;\cdots \;g_{q}[n]d_{H_{q}}[n]\;1\right]\times \left[\begin{array}{c} A_{q1}\\ \vdots \\ A_{qJ_{q}}\\ B_{q1}\\ \vdots \\ B_{qW_{q}}\\ -C_{q1}\\ \vdots \\ -C_{qH_{q}}\\ \phi_{q} \end{array}\right]+\eta_{q}\left[n\right]\\ & =\xi_{q}[n]\cdot \varphi_{q,G}+\tau_{q}[n],\;\mathrm{for}\;q=1,\ldots ,Q\;\mathrm{and}\;n=1,\ldots ,N. \end{array}$$

where $\xi_{q}[n]$ indicates the regression vector, which could be obtained from the microarray data and $\varphi_{q,G}$ denotes the unknown parameters vector for the *q*-th gene. We could expand the Equation (7) for *N* samples as shown below:

(8) $$\left[\begin{array}{c} g_{q}[1]\\ g_{q}[2]\\ \vdots \\ g_{q}[N] \end{array}\right]=\left[\begin{array}{c} \xi_{q,G}[1]\\ \xi_{q,G}[2]\\ \vdots \\ \xi_{q,G}[N] \end{array}\right]\cdot \varphi_{q,G}+\left[\begin{array}{c} \tau_{q}\left[1\right]\\ \tau_{q}\left[2\right]\\ \vdots \\ \tau_{q}\left[N\right] \end{array}\right]$$

Moreover, the Equation (8) could be simplified in the following form:

(9) $$G_{q}=\Xi_{q,G}\cdot \varphi_{q,G}+T_{q}$$

Hence, by solving the following constrained linear least square estimation problem, we could have the estimated regulatory parameters in the vector $\varphi_{q,G}$.

(10) $${\hat{\varphi }}_{q,G}=\underset{\varphi_{q,G}}{\min}\frac{1}{2}{\left\|\Xi_{q,G}\cdot \varphi_{q,G}-G_{q}\right\|}_{2}^{2}\;\mathrm{subject}\;\mathrm{to}\;\underset{J_{q}}{\left[\begin{array}{ccccc} 0 & \cdots & \cdots & 0 & 0\\ \vdots & \ddots & & \vdots & \vdots \\ \vdots & & \ddots & \vdots & \vdots \\ 0 & \cdots & \cdots & 0 & 0 \end{array}\right.}\underset{\;W_{q}}{\begin{array}{cccc} \cdots & \cdots & 0 & 1\\ \ddots & & \vdots & 0\\ & \ddots & \vdots & \vdots \\ \cdots & \cdots & 0 & 0 \end{array}}\underset{\;H_{q}}{\left.\begin{array}{cccc} 0 & \cdots & 0 & 0\\ \ddots & \ddots & \vdots & \vdots \\ \ddots & \ddots & 0 & \vdots \\ \cdots & 0 & 1 & 0 \end{array}\right]}\varphi_{q,G}\leq \left[\begin{array}{c} 0\\ \vdots \\ \vdots \\ 0 \end{array}\right]$$

where ${\hat{\varphi }}_{q,G}$ is the estimated vector including estimated regulatory parameters in the Equation (2). Meanwhile, the miRNA repression parameters $C_{qh}$ are guaranteed to be positive (i.e., $C_{qh}\geq 0$) for $h=1,\ldots ,H_{q}$.

It is noted that there are many false-positive interactions in the candidate GWGEN as a result of various experimental conditions in different databases. Here, we applied a system order detection approach in Equations (5) and (9) to prune the false-positive interactions. According to the Akaike information criterion (AIC) theory [101], the smallest AIC value would lead to the most accurate model. In other words, the smaller the AIC value we get, the closer we detect to the real system order. The formulas of AIC for determining the system order of interactions among the *i*-th protein and the *q*-th gene are given as below:

(11) $$\begin{array}{l} AIC(Y_{i})=\log({\hat{\rho }}_{i,P}^{2})+\frac{2(Y_{i}+1)}{N}\\ \mathrm{where}\;{\hat{\rho }}_{i,P}=\sqrt{\frac{{\left(P_{i}-(\Xi_{i,P}\cdot {\hat{\varphi }}_{i,P})\right)}^{T}(P_{i}-(\Xi_{i,P}\cdot {\hat{\varphi }}_{i,P}))}{N}} \end{array}$$

${\hat{\rho }}_{i,P}$ and $Y_{i}$ denote the estimated residual error and the number (system order) of PPIs with the *i*-th protein, respectively; ${\hat{\varphi }}_{i,P}$ denotes the estimated interaction parameters of the *i*-th protein by solving (6). Based on the AIC theory, the real system order $Y_{i}{}^{*}$ resulting in the smallest $AIC(Y_{i}{}^{*})$.

(12) $$\begin{array}{l} AIC(J_{q},W_{q},H_{q})=\log({\hat{\rho }}_{q,G}^{2})+\frac{2(\theta_{q,G}+1)}{N}\\ \mathrm{where}\;{\hat{\rho }}_{q,G}=\sqrt{\frac{{\left(G_{q}-(\Xi_{q,G}\cdot {\hat{\varphi }}_{q,G})\right)}^{T}(G_{q}-(\Xi_{q,G}\cdot {\hat{\varphi }}_{q,G}))}{N}}\;\mathrm{and}\;\theta_{q,G}=J_{q}+W_{q}+H_{q} \end{array}$$

${\hat{\rho }}_{i,P}$ and $\theta_{q,G}$ represent the estimated residual error and the number of regulations on the *q*-th gene, respectively; ${\hat{\varphi }}_{q,G}$ is the estimated parameter vector of the *q*-th gene obtained by solving (9). It is noted that the real system order $J_{q}{}^{*}+W_{q}{}^{*}+H_{q}{}^{*}$ lead to the smallest $AIC(J_{q}{}^{*}+W_{q}{}^{*}+H_{q}{}^{*})$. For each protein, gene, miRNA, and lncRNA, we used forward and backward search to find the real system order by AIC. The unimportant interactions among the candidate GWGEN, which are out of the system order, would be removed via the system order detection approach. By doing so, we could find the real GWGENs of DLBCL GCB and DLBCL ABC, respectively. The system identification method and system order detection approach could be applied to the lncRNA and miRNA system models as well (Supplementary Materials).

### 4.4. Extracting the Core GWGENs from the Real GWGENs by Principal Network Projection (PNP) Method

Although we have pruned the false-positive interactions from the candidate GWGEN by the system identification method and system order detection approach, the real GWGENs of DLBCL GCB and DLBCL ABC in Figures S1 and S2 are still too complex to investigate the common and specific pathogenic molecular mechanisms between DLBCL GCB and DLBCL ABC. Therefore, we utilize the PNP method to extract the core GWGENs from the real GWGENs of DLBCL GCB and DLBCL ABC. Before using the PNP method, we have to build a combined network matrix *Z* as follows:

(13) $$Z=\left[\begin{array}{ccccccccccccccc} {\hat{\alpha }}_{11} & \cdots & {\hat{\alpha }}_{1k} & \cdots & {\hat{\alpha }}_{1K} & 0 & \cdots & 0 & \cdots & 0 & 0 & \cdots & 0 & \cdots & 0\\ \vdots & \ddots & \vdots & \ddots & \vdots & \vdots & \ddots & \vdots & \ddots & \vdots & \vdots & \ddots & \vdots & \ddots & \vdots \\ {\hat{\alpha }}_{i1} & \cdots & {\hat{\alpha }}_{ik} & \cdots & {\hat{\alpha }}_{iK} & 0 & \cdots & 0 & \cdots & 0 & 0 & \cdots & 0 & \cdots & 0\\ \vdots & \ddots & \vdots & \ddots & \vdots & \vdots & \ddots & \vdots & \ddots & \vdots & \vdots & \ddots & \vdots & \ddots & \vdots \\ {\hat{\alpha }}_{I1} & \cdots & {\hat{\alpha }}_{Ik} & \cdots & {\hat{\alpha }}_{IK} & 0 & \cdots & 0 & \cdots & 0 & 0 & \cdots & 0 & \cdots & 0\\ {\hat{A}}_{11} & \cdots & {\hat{A}}_{1j} & \cdots & {\hat{A}}_{1J} & {\hat{B}}_{11} & \cdots & {\hat{B}}_{1w} & \cdots & {\hat{B}}_{1W} & -{\hat{C}}_{11} & \cdots & -{\hat{C}}_{1h} & \cdots & -{\hat{C}}_{1H}\\ \vdots & \ddots & \vdots & \ddots & \vdots & \vdots & \ddots & \vdots & \ddots & \vdots & \vdots & \ddots & \vdots & \ddots & \vdots \\ {\hat{A}}_{q1} & \cdots & {\hat{A}}_{qj} & \cdots & {\hat{A}}_{qJ} & {\hat{B}}_{q1} & \cdots & {\hat{B}}_{qw} & \cdots & {\hat{B}}_{qW} & -{\hat{C}}_{q1} & \cdots & -{\hat{C}}_{qh} & \cdots & -{\hat{C}}_{qH}\\ \vdots & \ddots & \vdots & \ddots & \vdots & \vdots & \ddots & \vdots & \ddots & \vdots & \vdots & \ddots & \vdots & \ddots & \vdots \\ {\hat{A}}_{Q1} & \cdots & {\hat{A}}_{Qj} & \cdots & {\hat{A}}_{QJ} & {\hat{B}}_{Q1} & \cdots & {\hat{B}}_{Qw} & \cdots & {\hat{B}}_{QW} & -{\hat{C}}_{Q1} & \cdots & -{\hat{C}}_{Qh} & \cdots & -{\hat{C}}_{QH}\\ {\hat{\beta }}_{11} & \cdots & {\hat{\beta }}_{1j} & \cdots & {\hat{\beta }}_{1J} & {\hat{\chi }}_{11} & \cdots & {\hat{\chi }}_{1w} & \cdots & {\hat{\chi }}_{1W} & -{\hat{\gamma }}_{11} & \cdots & -{\hat{\gamma }}_{1h} & \cdots & -{\hat{\gamma }}_{1H}\\ \vdots & \ddots & \vdots & \ddots & \vdots & \vdots & \ddots & \vdots & \ddots & \vdots & \vdots & \ddots & \vdots & \ddots & \vdots \\ {\hat{\beta }}_{v1} & \cdots & {\hat{\beta }}_{vj} & \cdots & {\hat{\beta }}_{vJ} & {\hat{\chi }}_{v1} & \cdots & {\hat{\chi }}_{vw} & \cdots & {\hat{\chi }}_{vW} & -{\hat{\gamma }}_{v1} & \cdots & -{\hat{\gamma }}_{vh} & \cdots & -{\hat{\gamma }}_{vH}\\ \vdots & \ddots & \vdots & \ddots & \vdots & \vdots & \ddots & \vdots & \ddots & \vdots & \vdots & \ddots & \vdots & \ddots & \vdots \\ {\hat{\beta }}_{V1} & \cdots & {\hat{\beta }}_{Vj} & \cdots & {\hat{\beta }}_{vJ} & {\hat{\chi }}_{v1} & \cdots & {\hat{\chi }}_{Vw} & \cdots & {\hat{\chi }}_{VW} & -{\hat{\gamma }}_{V1} & \cdots & -{\hat{\gamma }}_{Vh} & \cdots & -{\hat{\gamma }}_{VH}\\ {\hat{\sigma }}_{11} & \cdots & {\hat{\sigma }}_{1j} & \cdots & {\hat{\sigma }}_{1J} & {\hat{\delta }}_{11} & \cdots & {\hat{\delta }}_{1w} & \cdots & {\hat{\delta }}_{1W} & -{\hat{\omega }}_{11} & \cdots & -{\hat{\omega }}_{1h} & \cdots & -{\hat{\omega }}_{1H}\\ \vdots & \ddots & \vdots & \ddots & \vdots & \vdots & \ddots & \vdots & \ddots & \vdots & \vdots & \ddots & \vdots & \ddots & \vdots \\ {\hat{\sigma }}_{m1} & \cdots & {\hat{\sigma }}_{mj} & \cdots & {\hat{\sigma }}_{mJ} & {\hat{\delta }}_{m1} & \cdots & {\hat{\delta }}_{mw} & \cdots & {\hat{\delta }}_{mW} & -{\hat{\omega }}_{m1} & \cdots & -{\hat{\omega }}_{mh} & \cdots & -{\hat{\omega }}_{mH}\\ \vdots & \ddots & \vdots & \ddots & \vdots & \vdots & \ddots & \vdots & \ddots & \vdots & \vdots & \ddots & \vdots & \ddots & \vdots \\ {\hat{\sigma }}_{M1} & \cdots & {\hat{\sigma }}_{Mj} & \cdots & {\hat{\sigma }}_{MJ} & {\hat{\delta }}_{M1} & \cdots & {\hat{\delta }}_{Mw} & \cdots & {\hat{\delta }}_{MW} & -{\hat{\omega }}_{M1} & \cdots & -{\hat{\omega }}_{Mh} & \cdots & -{\hat{\omega }}_{MH} \end{array}\right]\in {ℜ}^{\left(I*+Q*+V*+M*)\times (J*+W*+H*\right)}$$

where the estimated parameters in (13) are obtained by solving the constrained linear least square estimation problem and conducting a system order detection approach based on AIC. The entry, which is pruned by AIC, would be padded with zero. The *i*-th row of *Z* denotes the interaction and regulation parameters of the *i*-th node in the real GWGEN. The PNP method is based on the singular value decomposition of *Z* shown as below:

(14) $$Z=UKG^{T}$$

where $U\in {ℜ}^{\left(I*+Q*+V*+M*)\times (I*+Q*+V*+M*\right)}$, $G\in {ℜ}^{{}^{\left(J*+W*+H*)\times (J*+W*+H*\right)}}$, $K=diag(k_{1},\cdots ,k_{r},\cdots ,k_{J*+W*+H*})\in {ℜ}^{\left(I*+Q*+V*+M*)\times (J*+W*+H*\right)}$ and *K* denotes the diagonal matrix which consists of J*+W*+H* singular values of *Z* in descending order (i.e., $k_{1}\geq \cdots \geq k_{r}\geq \cdots \geq k_{J^{*}+W^{*}+H^{*}}\geq 0$). The normalization of singular values is defined as below:

(15) $$E_{r}=\frac{k_{r}^{2}}{\sum_{r=1}^{J^{*}+W^{*}+H^{*}}k_{r}^{2}},\;\;\;\sum_{i=1}^{J^{*}+W^{*}+H^{*}}E_{r}^{}=1$$

Here, we choose the top *R* normalized singular values of combined network matrix *Z* with the minimum *R* to satisfy $\sum_{r=1}^{R}E_{r}^{}\geq 0.85$. It shows that we could use the top *R* singular vectors to construct 85% network structure as principal network structure. Afterwards, we project each node in the real GWGEN (i.e., each row in *Z*) to the top *R* singular vectors in $G^{T}$ as below:

(16) $$F(t,a)=d_{t,:}\cdot r_{a,:}^{T},\;\mathrm{for}\;t=1,\ldots I^{*}+Q^{*}+V^{*}+M^{*},\;a=1,\ldots R.$$

where $d_{t,:}$ denotes the *t*-th row vector of *Z*; $r_{a,:}^{T}$ is the *a*-th singular vector of $G^{T}$. Subsequently, we compute the 2-norm projection value for each node in the following:

(17) $$P(t)=\sqrt{\sum_{a=1}^{R}F^{2}(t,a)},\;\mathrm{for}\;t=1,\ldots I^{*}+Q^{*}+V^{*}+M^{*},\;a=1,\ldots R.$$

where P(t) denotes the 2-norm projection value of each *t*-th node in the real GWGEN on the top *R* singular vectors. The greater a projection value is, the more significant the *t*-th node in the principal structure of the real GWGEN. If the projection value approaches zero, it means that the related *t*-th node is almost independent to the principal network structure. In other words, the greater the projection value of a node in real GWGEN is, the higher probability is that a node will be an important component in the principal network structure. Lastly, the core GWGENs of DLBCL ABC and DLBCL GCB could be extracted from the real GWGENs based on the top-rank 3000 projection values of the nodes. Moreover, the core GWGENs of DLBCL ABC and DLBCL GCB are shown in Figures S3 and S4.

### 4.5. Deep Neural Netwok (DNN)-Based Drug-Target Interaction (DTI) Model for Multiple-Molecule Drug Design

To train a DNN-based DTI model, the drug-target interaction dataset came from BindingDB [102]. We picked drugs that at least had four interactions. Hence, in the selected dataset, there are 80,291 known drug-target interactions between 38,015 drugs and 7292 proteins. In order to simply avoid a class imbalance issue, which would degrade the training performance or make the learning progress biased toward the majority class, we randomly chose the negative instance (unknown drug-target pair) in the same size as positive instance (known drug-target pair). We trained the model using 70% of the data containing 10% of the data as validation set. The remaining 30% of data were used as testing set. Delineating the drug-target pair in a numerical vector, we transformed them into a feature vector by PyBioMed Python package under python 2.7 environment [103]. The PyMolecule module in PyBioMed was responsible to transform the drug descriptors. The drug features include commonly used structural and physicochemical information. The PyProtein module in PyBioMed was applied to transform the target descriptors. The target features were computed based on the structural and physicochemical properties of proteins and peptide from amino acid sequence. The feature vector for each drug-target pair can be represented in the following form:

(18) $$w_{drug-target}=\left[D,T\right]=\left[d_{1},d_{2},\cdots ,d_{X},t_{1},t_{2},\cdots ,t_{Y}\right]$$

where $w_{drug-target}$ denotes a feature vector of drug-target pair, *X* and *Y* are the total number of drug features and target features, which are 363 and 996, respectively; *D* and *T* indicates the feature vector of the relevant drug-target pair; $d_{X}$ is the *x*-th drug feature and $t_{Y}$ is the *y*-th target feature. Since the drug and target features are measured in different scales, we performed normalization before training. Then, we applied principal component analysis (PCA) [104] to decrease the feature size from 1359 to 618. By doing so, we not only could remove noisy feature but also reduce memory consumption.

For the architecture of the DNN-based DTI model, the input layer contains 618 neurons, followed 512, 256, 128, and 64 neurons in the hidden layers, respectively. The output layer is with one neuron. The optimal hyperparameters were found based on 10-fold cross validation (Figure S6). Each layer of DNN-based DTI model could be simplified into a function as follows:

(19) $$h_{n}=\sigma (w^{T}x_{n}+b)$$

where $x_{n}$ denotes the input of the *n*-th drug-target feature vector, $h_{n}$ indicates the output of each layer; w is the weighting matrix; b is the bias vector; σ is the activation function, by which sigmoid activation function is used for the output layer and ReLU [105] is used for the hidden layer. We added dropout on each hidden layer for reducing overfitting. Meanwhile, the model training would be terminated once the model performance stopped to improve on the validation set by early stopping function. Moreover, we chose the binary cross-entropy to be the cost function:

(20) $$\begin{array}{l} C_{n}(w,b)=-\frac{1}{N}\sum_{n=1}^{N}(p_{n}\log({\hat{p}}_{n})+(1-p_{n})\log(1-{\hat{p}}_{n}))\\ L(w,b)=\frac{1}{N}\sum_{n=1}^{N}C_{n}(p_{n},{\hat{p}}_{n}) \end{array}$$

where L(w,b) is the average of total loss; $p_{n}$ denotes the *n*-th true positive instance (1) or true negative instance (0) of drug-target binding; ${\hat{p}}_{n}$ denotes the *n*-th predicted probability of positive instance (1) or predicted probability of negative instance (0) of drug-target binding. For obtaining the optimal network parameter set $\phi^{*}$, the cost function is in the following:

(21) $$\phi^{*}=\arg\;\underset{\phi }{\min}\;L(\phi )$$

The above equation could be achieved by the backpropagation algorithm [106]. The updated weight and bias parameters for the *j*-th epoch is shown as below:

(22) $$\begin{array}{l} \phi^{j}=\phi^{j-1}-\eta \nabla L(\phi^{j-1}),\\ \mathrm{where}\;\nabla L(\phi^{j-1})=\left[\begin{array}{c} \frac{\partial L(\phi^{j-1})}{\partial w_{1}}\\ \vdots \\ \frac{\partial L(\phi^{j-1})}{\partial w_{h}}\\ \frac{\partial L(\phi^{j-1})}{\partial b_{1}}\\ \vdots \\ \frac{\partial L(\phi^{j-1})}{\partial b_{h}} \end{array}\right]. \end{array}$$

where η is the learning rate, which is 0.001; $\nabla L(\phi^{j-1})$ denotes the gradient of $L(\phi^{j-1})$.

## Figures and Tables

**Table 1:** The biomarkers (drug targets) are identified for DLBCL ABC and DLBCL GCB.

Cancer	Biomarkers (Drug Targets)
DLBCL ABC	FOXL1 NFκB1 AKT1 MYC STAT3
DLBCL GCB	FOXL1 NFκB1 AKT1 MYC EZH2

**Table 2:** The multiple-molecule drug and the corresponding target proteins for DLBCL ABC.

	Targets	FOXL1	NFκB1	AKT1	MYC	STAT3
Drugs						
Famotidine			O	O		O
Chlorzoxazone		O	O			O
Etoposide			O		O	O

O: The drug targets to its potential target proteins.

**Table 3:** The multiple-molecule drug and the corresponding target proteins for DLBCL GCB.

	Targets	FOXL1	NFκB1	AKT1	MYC	EZH2
Drugs						
Famotidine			O	O		O
Chlorzoxazone		O	O			O
Methotrexate				O	O	O

O: The drug targets to its potential target proteins.

## Data Availability

The DLBCL microarray data is from GSE117556 (https://www.ncbi.nlm.nih.gov/geo/query/acc.cgi?acc=GSE117556, accessed on 18 May 2022).

## Supplementary Materials

The following supporting information can be downloaded at: https://www.mdpi.com/article/10.3390/ijms23126732/s1.
