# Supplementary material for: Systems Drug Discovery for Diffuse Large B Cell Lymphoma Based on Pathogenic Molecular Mechanism via Big Data Mining and Deep Learning Method
Source: Int J Mol Sci. 2022 Jun 16;23(12):6732. doi: 10.3390/ijms23126732 (PMC9224183; doi:10.3390/ijms23126732)
Supplement: Supplementary file 1 [file ijms-23-06732-s001.zip › ijms-1757272-supplementary/suppl/Supplementary_r1.pdf]

Supplementary

1.1 Systems modeling of lncRNAs and miRNAs

The expression level of lncRNA might be influenced by the regulations of TFs, lncRNA and miRNAs. Hence, the candidate lncRNA regulatory model of candidate GENs by the regulatory equations in sample n is described as follows:

$$s_v[n] = \sum_{j=1}^{J_v} \beta_{vj} z_j[n] + \sum_{\substack{w=1 \\ w \neq v}}^{W_v} \chi_{vw} x_w[n] - \sum_{h=1}^{H_v} \gamma_{vh} d_h[n] s_v[n] + \phi_v + \eta_v[n] \quad \text{(S1)}$$

for $v = 1, \ldots, V$ and $n = 1, \ldots N$.

where $s_v[n]$ represents the expression level of the v-th lncRNA; J_v indicates the total number of TFs binding to the v-th lncRNA; W_v represents the total number of lncRNAs binding to the v-th lncRNA; H_v denotes the total number of miRNAs inhibiting the v-th lncRNA; β_{vj} denotes the transcription regulatory ability from the j-th TF to the v-th lncRNA; χ_{vw} is the regulation ability from the w-th lncRNA to the v-th lncRNA; $\gamma_{vh} \geq 0$ represents the post-transcriptional regulation ability with which the h-th miRNA inhibits the v-th lncRNA; $z_j[n]$, $x_w[n]$, and $d_h[n]$ indicate the expression of the j-th TF, the w-th lncRNA, and the h-th miRNA, respectively. V is the total number of lncRNAs and N denotes the total number of patient samples; ϕ_v represents the basal level of the v-th lncRNA coming from unknown regulations; $\eta_v[n]$ is the stochastic noise of the v-th lncRNA for the sample n caused by the modeling residue and data measurement noise.

Similarly, for the candidate miRNA regulation network (MRN) in the candidate GWGEN, the systematic regulation model is defined as below:

$$l_m[n] = \sum_{j=1}^{J_m} \sigma_{mj} z_j[n] + \sum_{w=1}^{W_m} \delta_{mw} x_w[n] - \sum_{\substack{h=1 \\ h \neq m}}^{H_m} \omega_{mh} d_h[n] l_m[n] + \phi_m + \eta_m[n] \quad \text{(S2)}$$

for $m = 1, \ldots, M$ and $n = 1, \ldots N$.

where $l_m[n]$ represents the expression level of the m-th miRNA; J_m indicates the total number of TFs binding to the m-th miRNA; W_m represents the total number of lncRNAs binding to the m-th miRNA; H_m denotes the total number of miRNAs inhibiting the m-th miRNA; σ_{mj} denotes the transcription regulatory ability from the j-th TF to the m-th miRNA; δ_{mw} is the regulation ability from the w-th lncRNA to the

m-th miRNA; $\omega_{mh} \geq 0$ represents the post-transcription regulatory ability with which the h-th miRNA inhibits the v-th miRNA; $z_j[n]$, $x_w[n]$, and $d_h[n]$ indicate the expression of the j-th TF, the w-th lncRNA, and the h-th miRNA, respectively. M is the total number of miRNAs and N denotes the total number of patient samples; ϕ_m represents the basal level of the z-th miRNA due to some unknown regulations; $\eta_m[n]$ is the stochastic noise of the m-th miRNA for the sample n owing to the modeling residue and data noise.

1.2 System identification and system order detection approach to lncRNA and miRNA system models

To estimate the unknown parameters for the lncRNA model in the candidate GWGEN, the lncRNA equation in equation (S1) could be rewritten as below:

$$s_v[n] = \begin{bmatrix} z_1[n] & \cdots & z_{J_v}[n] & x_1[n] & \cdots & x_{W_v}[n] & d_1[n]s_v[n] & \cdots & d_{H_v}[n]s_v[n] & 1 \end{bmatrix} \times \begin{bmatrix} \beta_{v1} \\ \vdots \\ \beta_{vJ_v} \\ \chi_{v1} \\ \vdots \\ \chi_{vW_v} \\ -\gamma_{v1} \\ \vdots \\ -\gamma_{vH_v} \\ \phi_v \end{bmatrix} + \eta_v[n]$$
$$= \xi_v[n] \cdot \varphi_{v,L} + \tau_v[n], \text{ for } s = 1, \ldots, L \text{ and } n = 1, \ldots, N. \tag{S3}$$

Where $\xi_v[n]$ determines the regression vector which could be computed by the microarray data; $\varphi_{v,L}$ indicates the unknown parameter vector for the v-th lncRNA. The equation (S3) of the v-th lncRNA could be augmented for N samples as below:

$$\begin{bmatrix} s_v[1] \\ s_v[2] \\ \vdots \\ s_v[N] \end{bmatrix} = \begin{bmatrix} \xi_{v,L}[1] \\ \xi_{v,L}[2] \\ \vdots \\ \xi_{v,L}[N] \end{bmatrix} \cdot \varphi_{v,L} + \begin{bmatrix} \tau_v[1] \\ \tau_v[2] \\ \vdots \\ \tau_v[N] \end{bmatrix} \tag{S4}$$

Furthermore, we simplified (S4) to the following form:

$$S_v = \Xi_{v,L} \cdot \varphi_{v,L} + \mathrm{T}_v \tag{S5}$$

Therefore, the unknown parameters in the vector $\varphi_{v,L}$ could be estimated by solving

the constrained liner least square estimation problem:

$$\hat{\varphi}_{v,L} = \min_{\varphi_{v,L}} \frac{1}{2}\left\| \Xi_{v,L} \cdot \varphi_{v,L} - S_v \right\|_2^2$$

$$\text{subject to } \underbrace{\begin{bmatrix} 0 & \cdots & \cdots & 0 \\ \vdots & \ddots & & \vdots \\ \vdots & & \ddots & \vdots \\ 0 & \cdots & \cdots & 0 \end{bmatrix}}_{J_v} \begin{matrix} \\ \\ \\ \end{matrix} \left[\begin{array}{cccc|cccc|cccc|c} 0 & \cdots & \cdots & 0 & 0 & \cdots & \cdots & 0 & 1 & 0 & \cdots & 0 & 0 \\ \vdots & \ddots & & \vdots & \vdots & \ddots & & \vdots & 0 & \ddots & \ddots & \vdots & \vdots \\ \vdots & & \ddots & \vdots & \vdots & & \ddots & \vdots & \vdots & \ddots & \ddots & 0 & \vdots \\ 0 & \cdots & \cdots & 0 & 0 & \cdots & \cdots & 0 & 0 & \cdots & 0 & 1 & 0 \end{array}\right] \varphi_{v,L} \le \begin{bmatrix} 0 \\ \vdots \\ \vdots \\ 0 \end{bmatrix} \quad \text{(S6)}$$

(column blocks: J_v, M_v, H_v)

where $\hat{\varphi}_{v,L}$ is the estimated vector consisting of the estimated regulatory parameters in the equation (S1). Meanwhile, the miRNA repression parameters γ_{vh} are guaranteed to be positive (i.e. $\gamma_{vh} \ge 0$) for $h = 1, \ldots, H_v$.

Similarly, the miRNA regulation model in equation (S2) could be rewritten as below:

$$l_m[n] = \left[z_1[n] \; \cdots \; z_{J_m}[n] \; x_1[n] \; \cdots \; x_{W_m}[n] \; d_1[n]\, l_m[n] \; \cdots \; d_{H_m}[n]\, l_m[n] \; 1 \right] \times \begin{bmatrix} \sigma_{m1} \\ \vdots \\ \sigma_{mJ_m} \\ \delta_{m1} \\ \vdots \\ \delta_{mW_m} \\ -\omega_{m1} \\ \vdots \\ -\omega_{mH_m} \\ \phi_m \end{bmatrix} + \eta_m[n] \quad \text{(S7)}$$

$$= \xi_m[n] \cdot \varphi_{m,M} + \tau_m[n], \text{for } m = 1, \ldots, M \text{ and } n = 1, \ldots, N.$$

Where $\xi_m[n]$ indicates the regression vector which could be obtained from the microarray data and $\varphi_{m,M}$ denotes the unknown parameters vector for the *m*-th miRNA. We could expand the equation (S7) for *N* samples as shown below:

$$\begin{bmatrix} l_m[1] \\ l_m[2] \\ \vdots \\ l_m[N] \end{bmatrix} = \begin{bmatrix} \xi_{i,M}[1] \\ \xi_{i,M}[2] \\ \vdots \\ \xi_{i,M}[N] \end{bmatrix} \cdot \varphi_{i,P} + \begin{bmatrix} \tau_m[1] \\ \tau_m[2] \\ \vdots \\ \tau_m[N] \end{bmatrix} \quad \text{(S8)}$$

Moreover, the equation (S8) could be simplified in the following form:

$$L_m = \Xi_{\mathrm{m},M} \cdot \varphi_{\mathrm{m},M} + \mathrm{T}_m \tag{S9}$$

Hence, by solving the following constrained linear least square estimation problem, we could have the estimated regulatory parameters in the vector $\varphi_{\mathrm{m},M}$.

$$\hat{\varphi}_{\mathrm{m},M} = \min_{\varphi_{\mathrm{m},M}} \frac{1}{2} \left\| \Xi_{\mathrm{m},M} \cdot \varphi_{\mathrm{m},M} - L_m \right\|_2^2 \tag{S10}$$

$$\text{subject to} \; \left[\begin{array}{cccc|cccc|cccc|c} 0 & \cdots & \cdots & 0 & 0 & \cdots & \cdots & 0 & 1 & 0 & \cdots & 0 & 0 \\ \vdots & \ddots & & \vdots & \vdots & \ddots & & \vdots & 0 & \ddots & \ddots & \vdots & \vdots \\ \vdots & & \ddots & \vdots & \vdots & & \ddots & \vdots & \vdots & \ddots & \ddots & 0 & \vdots \\ 0 & \cdots & \cdots & 0 & 0 & \cdots & \cdots & 0 & 0 & \cdots & 0 & 1 & 0 \end{array}\right] \varphi_{\mathrm{m},M} \le \begin{bmatrix} 0 \\ \vdots \\ \vdots \\ 0 \end{bmatrix}$$

$$\qquad\qquad \mathrm{J}_m \qquad\qquad\qquad \mathrm{W}_m \qquad\qquad\qquad \mathrm{H}_m$$

where $\hat{\varphi}_{\mathrm{m},M}$ is the estimated vector containing estimated regulatory parameters in the equation (S2). Meanwhile, the miRNA repression parameters ω_{mh} are guaranteed to be positive (i.e. $\omega_{mh} \ge 0$) for $h = 1, \ldots, H_m$.

The derived AIC formulas for the v-th lncRNA (S11) and the m-th miRNA (S12) are given as below:

$$\begin{aligned} &\mathrm{AIC}(\mathrm{J}_v,\mathrm{W}_v,\mathrm{H}_v)=\log(\hat{\rho}_{\mathrm{v,M}}^2)+\frac{2(\theta_{\mathrm{v,M}}+1)}{\mathrm{N}} \\ &\text{where } \hat{\rho}_{\mathrm{z,M}}=\sqrt{\frac{(\mathrm{S}_v\text{-}(\Xi_{\mathrm{v,M}}\cdot\hat{\varphi}_{\mathrm{v,M}}))^{\mathrm{T}}(\mathrm{S}_v\text{-}(\Xi_{\mathrm{v,M}}\cdot\hat{\varphi}_{\mathrm{v,M}}))}{\mathrm{N}}} \;,\; \theta_{\mathrm{v,M}}=\mathrm{J}_v+\mathrm{W}_v+\mathrm{H}_v \end{aligned} \tag{S11}$$

$\hat{\rho}_{\mathrm{z,M}}$ and $\theta_{\mathrm{v,M}}$ represent the estimated residual error and the number of the regulations on the v-th lncRNA, respectively; $\hat{\varphi}_{\mathrm{v,M}}$ is the estimated parameter vector of the v-th lncRNA by solving (S5). Based on the AIC theory, the real system order $J_v^* + W_v^* + H_v^*$ resulting in the smallest $AIC(J_v^* + W_v^* + H_v^*)$.

$$\begin{aligned} &AIC(J_m, W_m, H_m) = \log(\hat{\rho}_{m,M}^2) + \frac{2(\theta_{m,M}+1)}{N} \\ &\text{where } \hat{\rho}_{m,M} = \sqrt{\frac{(L_m - (\Xi_{m,M}\cdot\hat{\varphi}_{m,M}))^T (L_m - (\Xi_{m,M}\cdot\hat{\varphi}_{m,M}))}{N}} \;,\; \theta_{m,M} = J_m + W_m + H_m \end{aligned} \tag{S12}$$

$\hat{\rho}_{m,M}$ and $\theta_{m,M}$ represent the estimated residual error and the number of the regulations on the m-th miRNA, respectively; $\hat{\varphi}_{m,M}$ is the estimated parameter vector of the m-th miRNA by solving (S9). Based on the AIC theory, the real system order $J_m^* + W_m^* + H_m^*$ lead to the smallest $AIC(J_m^* + W_m^* + H_m^*)$.

Tables.

Table S1. The statistics of nodes and edges in the candidate GWGEN and the identified real GWGENs of DLBCL ABC and DLBCL GCB.

Node/Edge	Candidate GWGEN	DLBCL ABC Real GWGEN	DLBCL GCB Real GWGEN
LncRNA-LncRNA	2	1	1
LncRNA-TF	199	65	71
LncRNA-Protein	922	407	454
LncRNA node	259	221	228
LncRNA edge	1216	515	574
MiRNA-LncRNA	240	86	114
MiRNA-MiRNA	54	20	17
MiRNA-TF	17892	4077	4794
MiRNA-Protein	148606	65234	73190
MiRNA node	498	498	498
MiRNA edge	188374	78243	88037
TF-LncRNA	276	194	213
TF-MiRNA	2107	1908	1979
TF-TF	11839	6743	7918
TF-Protein	103024	79021	84526
TF node	798	729	749
TF edge	133731	100339	108081
Receptor node	2452	2457	2455
Protein node	15347	15187	15193
PPI edge	3997005	901174	911806
Total node	19354	19092	19123
Total edge	4320803	1080624	1108843

Table S2. The gene enrichment analysis results based on the genes in the core GWGEN of DLBCL ABC by DAVID.

Pathway enrichment	Numbers of proteins	p-value
Pathways in cancer	109	1.40E-06
PI3K-Akt signaling pathway	88	2.90E-04
Chemokine signaling pathway	63	1.80E-07
Toll-like receptor signaling pathway	40	4.80E-07
NF-kappa B signaling pathway	32	1.50E-05

Table S3. The gene enrichment analysis results based on the genes in the core GWGEN of DLBCL GCB by DAVID.

Pathway enrichment	Numbers of proteins	p-value
Pathways in cancer	108	4.10E-08
PI3K-Akt signaling pathway	85	1.20E-04
Chemokine signaling pathway	62	3.70E-08
MAPK signaling pathway	62	1.30E-03
T cell receptor signaling pathway	35	6.60E-05

Table S4. The drug candidates with toxicity and drug regulation ability information toward their corresponding targets.

**FOXL1**			**NFκB1**		
Drug	Toxicity (LD50,mol/kg)	Regulability (CMap)	Drug	Toxicity (LD50,mol/kg)	Regulability (CMap)
chlorzoxazone	2.2388	0.43578	famotidine	1.9523	-0.15561
cefmetazole	2.2638	0.407316	dacarbazine	1.9602	-0.09832
nizatidine	2.435	0.225334	chlorzoxazone	2.2388	-0.09968
ondansetron	2.4555	0.338226	etoposide	2.9588	-0.01479
**AKT1**			**MYC**		
Drug	Toxicity (LD50,mol/kg)	Regulability (CMap)	Drug	Toxicity (LD50,mol/kg)	Regulability (CMap)
famotidine	1.9523	-0.31407	dacarbazine	1.9602	-0.35287
dacarbazine	1.9602	-0.38088	paclitaxel	2.4391	-0.32761
clenbuterol	2.6024	-0.41223	etoposide	2.9588	-0.37365
ciclosporin	2.8788	-0.20654	sulfinpyrazone	3.0218	-0.34322
methotrexate	3.4955	-0.39957	methotrexate	3.4955	-0.37873

**STAT3**			**EZH2**		
Drug	Toxicity (LD50,mol/kg)	Regulability (CMap)	Drug	Toxicity (LD50,mol/kg)	Regulability (CMap)
dacarbazine	1.9602	-0.448	famotidine	1.9523	-0.4045
chlorzoxazone	2.2388	-0.077	chlorzoxazon e	2.2388	-0.2908
famotidine	2.9503	-0.462	chloroquine	2.9547	-0.4260
etoposide	2.9588	-0.4939	methotrexate	3.4955	-0.4870
clonidine	3.503	-0.5041	clonidine	3.5030	-0.4302

Figures.

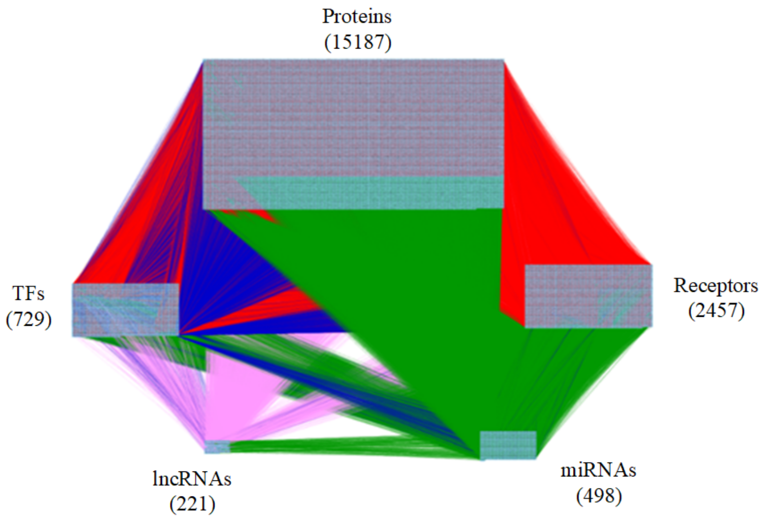

Figure S1. The real genome-wide genetic and epigenetic network (GWGEN) of DLBCL ABC. The red lines indicate protein-protein interactions (PPIs). The blue lines denote transcriptional regulations by TFs. The pink lines denote post-transcriptional regulations by lncRNAs; The green lines represent post-transcriptional regulations by miRNAs. The numbers of receptors, proteins, lncRNAs, TFs and miRNAs are 2546, 14149, 106, 1783 and 508, respectively.

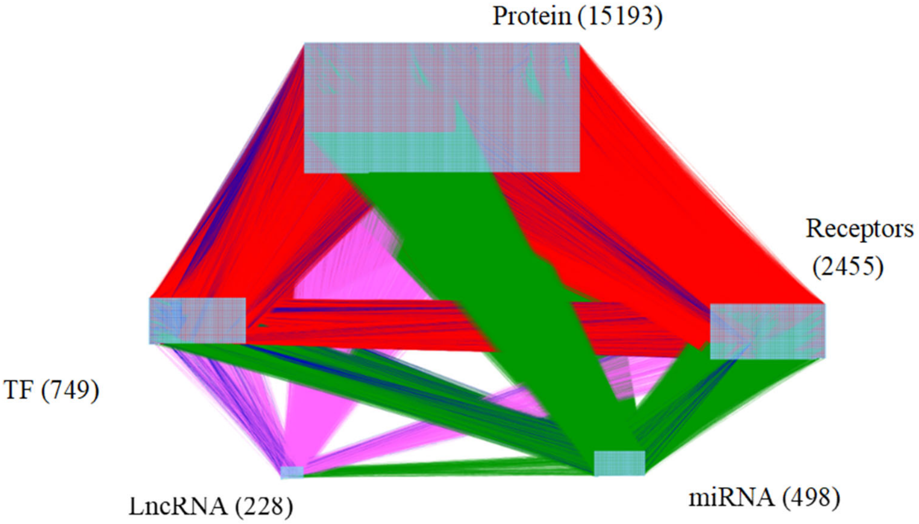

Figure S2. The real genome-wide genetic and epigenetic network (GWGEN) of DLBCL GCB. The red lines indicate protein-protein interactions (PPIs). The blue lines denote transcriptional regulations by TFs. The pink lines denote post-transcriptional regulations by lncRNAs. The green lines represent post-transcriptional regulations by miRNAs. The numbers of receptors, proteins, lncRNAs, TFs and miRNAs

are 2546, 14189, 111, 1769 and 508, respectively.

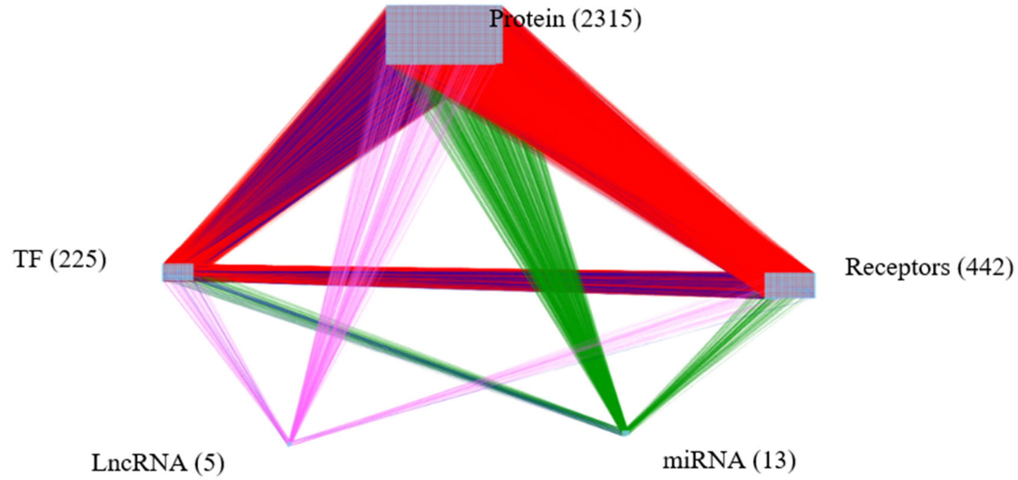

Figure S3. The core genome-wide genetic and epigenetic network (GWGEN) of DLBCL ABC. The red lines indicate protein-protein interactions (PPIs). The blue lines denote transcriptional regulations by TFs. The pink lines denote post-transcriptional regulations by lncRNAs. The green lines represent post-transcriptional regulations by miRNAs. The numbers of receptors, proteins, lncRNAs, TFs and miRNAs are 442, 2315, 5, 225 and 13, respectively.

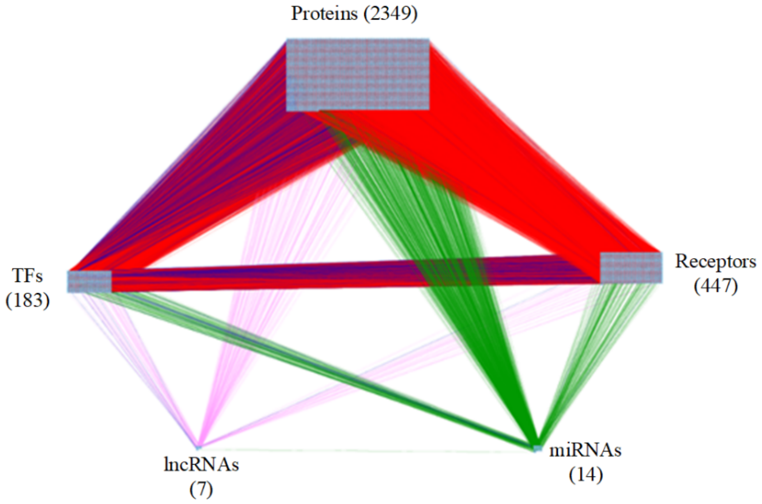

Figure S4. The core genome-wide genetic and epigenetic network (GWGEN) of DLBCL GCB. The red lines indicate protein-protein interactions (PPIs). The blue lines denote transcriptional regulations by TFs. The pink lines denote post-transcriptional regulations by lncRNAs. The green lines represent post-transcriptional regulations by miRNAs. The numbers of receptors, proteins, lncRNAs, TFs and miRNAs are 447, 2349, 7, 2349 and 14, respectively.

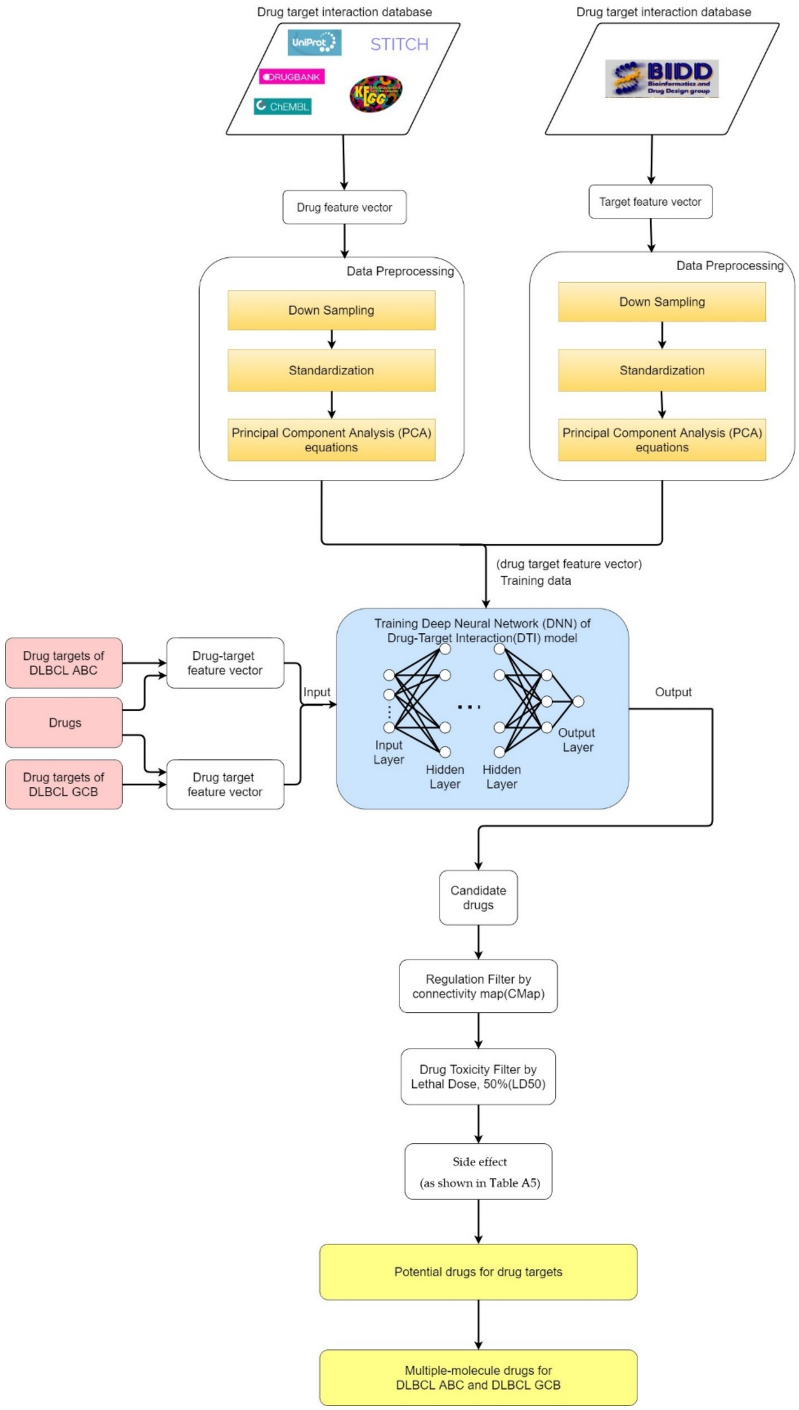

Figure S5. The systems drug design procedure.

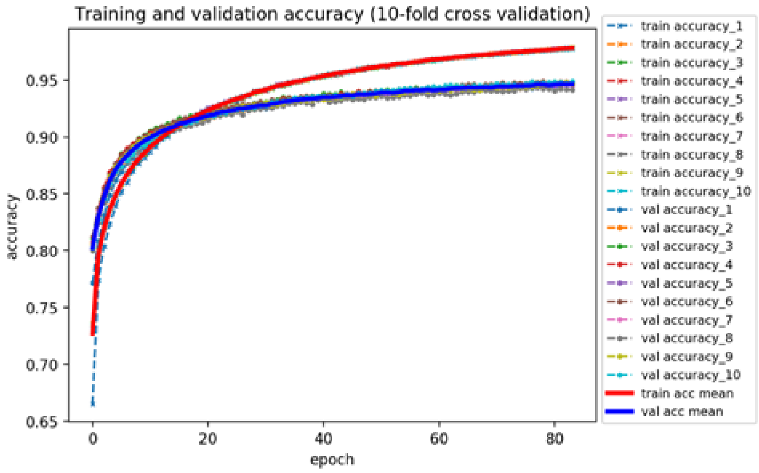

(A)

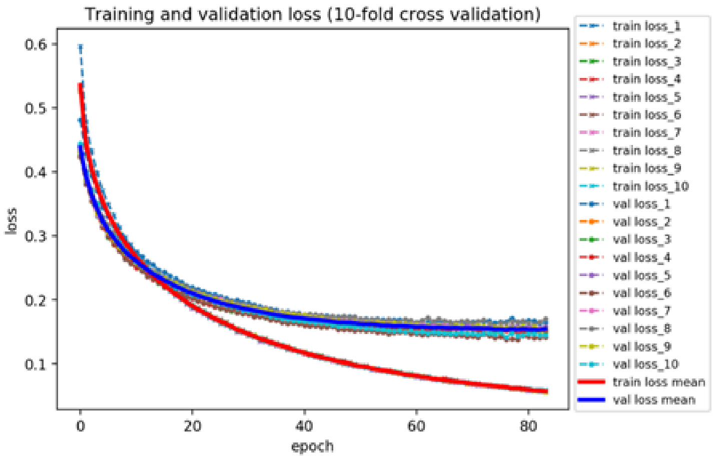

(B)

Figure S6. Training and Validation Learning curves (10-fold cross validation). (A)Training and validation accuracy. (B) Training and validation loss. "-x-" lines in different colors denote the training accuracy and loss, while "-o-" lines in different colors represent the validation accuracy and loss. The bold lines in red and blue indicate the model's average loss or accuracy of training and validation, respectively.

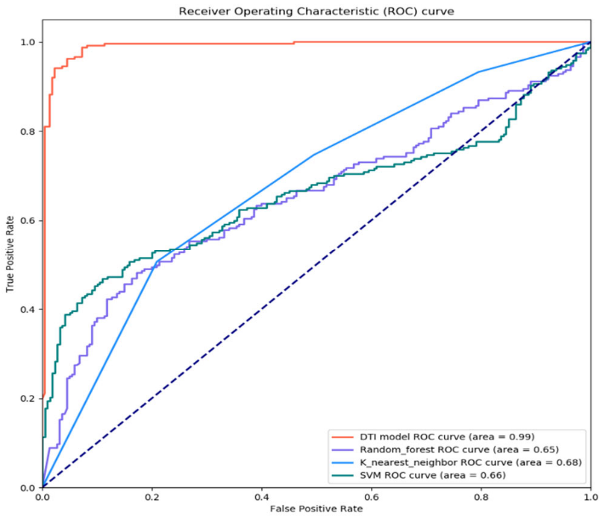

Figure S7. The ROC curves of different kinds of DTI models. This figure shows that the DTI model trained by DNN can achieve an AUC of 0.99, which is much better than the AUC of 0.65 by the random forest, much higher than the AUC of 0.68 by Nearest neighbor and much higher than the AUC of 0.66 by SVM. The results reflect that deep learning method is better than traditional machine learning methods. It is worth noting that the dot line is the case of AUC 0.5 as a result of the classifier following random prediction, therefore the model has no predictive value.
